# Synergistic Effects of Garlic Extract and Mannan‐Oligosaccharide Prebiotic Supplementation on Growth Performance, Carcass Quality, Immunity, Gut Morphology and Microbiome in Broiler Chickens

**DOI:** 10.1002/vms3.70751

**Published:** 2026-01-06

**Authors:** Azizullah Khan, Muhammad Mushtaq, Muqadar Shah, Rifat Ullah Khan, Rasha Alonaizan, Shabana Naz, Ala Abudabos, Muhammad Israr

**Affiliations:** ^1^ Department of Poultry Science Faculty of Animal Husbandry and Veterinary Sciences The University of Agriculture Peshawar Peshawar Pakistan; ^2^ College of Veterinary Sciences Faculty of Animal Husbandry and Veterinary Sciences The University of Agriculture Peshawar Peshawar Pakistan; ^3^ Department of Zoology College of Sciences King Saud University Riyadh Saudi Arabia; ^4^ Department of Zoology Government College University Faisalabad Pakistan; ^5^ Department of Food and Animal Sciences College of Agriculture Tennessee State University Nashville Tennessee USA; ^6^ Health Task London UK

**Keywords:** alternative growth promotors, feed additives, gut health, humoral immune response, weight gain

## Abstract

This study evaluated the effects of dietary supplementation with garlic extract, mannan oligosaccharide (MOS) and their combinations on growth performance, immunity, gut morphology and microbiota in broilers. A total of 250 Hubbard chicks were allocated into five groups: control (basal diet), garlic extract, MOS, Combo‐I (half doses of both) and Combo‐II (full doses of both). The trial lasted 42 days under standard management conditions. Results demonstrated that Combo‐I consistently improved body weight gain and feed conversion ratio (FCR) during the starter, grower and finisher phases (*p* < 0.05), highlighting a synergistic effect of the combined supplements. Carcass weight was significantly enhanced in Combo‐II (*p* = 0.03), although dressing percentage and organ weights were not affected (*p* > 0.05). Immunological parameters were strongly influenced by supplementation. Combo‐I induced the highest antibody titres against Newcastle disease and infectious bursal disease (*p* < 0.05), with Combo‐II ranking second, whereas single additives showed moderate improvements compared to the control. Similarly, serum immunoglobulin concentrations (IgM, IgA and IgG) were greatest in Combo‐I, confirming enhanced humoral immunity. Gut morphology was significantly improved in the combination groups, particularly Combo‐I, which showed increased villus height, wider villi, reduced crypt depth, and the highest villus:crypt ratio (*p* < 0.05), reflecting superior absorptive potential. Although differences in ileal microbiota were not statistically significant, both combination treatments reduced *Escherichia coli* and *Salmonella* counts while promoting *Lactobacillus* spp. populations. In conclusion, the combined use of garlic extract and MOS, especially at half doses (Combo‐I), optimally improved growth, immune response, gut architecture and microbial balance, offering a synergistic strategy for enhancing broiler performance.

## Graphical abstract

Garlic extract and mannan oligosaccharide (MOS) synergistically improved broiler growth, carcass weight, immune response, gut morphology and microbial balance, with half‐dose combination (Combo‐I) showing the most consistent benefits, offering a sustainable alternative to antibiotic growth promoters.

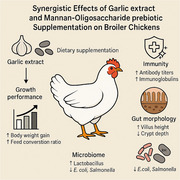

## 1 Introduction

The global poultry industry plays a vital role in ensuring food security by providing a cost‐effective source of high‐quality animal protein (Rahman et al. 2017; Chand et al. 2018; Dinasarki et al. 2024). Poultry production not only contributes to human nutrition but also supports the livelihoods of millions of small‐ and large‐scale farmers worldwide (Hafeez et al. 2020; Gul et al. 2024; Iqbal et al. 2024). In recent decades, advances in genetics, nutrition and management have significantly improved broiler productivity (Al‐Suwailem et al. 2024). However, the intensive nature of modern poultry farming has led to increased susceptibility to infectious diseases and environmental stressors, which can negatively affect bird health and performance (Othman et al. 2024). The overreliance on antibiotic growth promoters (AGPs) has raised concerns about antibiotic resistance, prompting regulatory restrictions and a search for safer, sustainable alternatives (Othman et al. 2024). Among the promising alternatives, prebiotics, probiotics and their combinations (synbiotics) have received significant attention for their ability to enhance gut health, immune function and overall growth performance in poultry (Magnoli et al. 2024; Tangthirasunun et al. 2024). These feed additives can modulate the intestinal microbiota, improve nutrient digestibility and reduce the incidence of enteric infections, thus supporting sustainable and antibiotic‐free poultry production (Ciptaan et al. 2024; Habib et al. 2024; Usman et al. 2024).

Garlic is a well‐known herb containing a wide array of bioactive compounds, including allicin, diallyl sulphides and ajoene, which exhibit strong antimicrobial, antioxidant and immunostimulatory properties (Abd El‐Ghany 2024). These compounds can inhibit the growth of harmful intestinal bacteria while promoting beneficial microflora, contributing to improved gut health and nutrient absorption (Amagase 2006; Basit et al. 2025). In broilers, garlic supplementation has been shown to enhance feed intake, body weight gain (BWG), feed conversion efficiency and immunity (Khan et al. 2012). Moreover, garlic's antioxidant properties can help mitigate oxidative stress in broilers, which is particularly important under intensive production conditions or environmental stress (Hafeez et al. 2024). Additionally, garlic improves intestinal morphology by increasing villus height and reducing crypt depth (CD), which enhances the absorptive surface area of the small intestine and facilitates efficient nutrient uptake (Khan et al. 2012). Prebiotics are nondigestible oligosaccharides that selectively stimulate the growth and activity of beneficial gut bacteria, such as *Lactobacillus* and *Bifidobacterium*, thereby improving intestinal integrity and nutrient absorption (Alqahtani et al. 2024; Dabool et al. 2024; Yu et al. 2024). In addition to promoting gut microbiota balance, prebiotics can enhance immune responsiveness by stimulating gut‐associated lymphoid tissues (GALT) and modulating systemic immunity. When combined, phytogenic and prebiotic may exert synergistic effects on intestinal morphology, immune modulation and overall growth performance by improving gut microbial balance, enhancing digestive efficiency and stimulating local immune responses (Borgohain et al. 2017; Shehata et al. 2022). This combined approach represents a sustainable strategy to optimize broiler productivity while reducing reliance on antibiotics, thereby addressing both production efficiency and food safety concerns.

The objective of this study was to evaluate the individual and combined effects of dietary garlic extract and prebiotic on growth performance, immune response and intestinal morphology of broiler chickens. The selected levels were based on previously published studies that demonstrated effective and safe dosages of mannan oligosaccharide (MOS) and garlic extract in poultry diets. Specifically, 200 mg/kg MOS and 60 mg/kg garlic extract have been reported to improve growth performance, gut health and immune response without adverse effects. To evaluate potential synergistic effects, we also included a half‐dose combination (Combo‐I) and a full‐dose combination (Combo‐II), allowing us to compare the efficacy of reduced and full supplementation levels.

## 2 Materials and Methods

### 2.1 Broiler Rearing and Experimental Treatments

A total of 250 day‐old broiler (Hubbard) chicks were obtained from a local commercial hatchery. Chicks with uniform body weight were carefully selected and randomly allocated into five treatment groups. Each group was further divided into five replicates, comprising 10 chicks per replicate. The birds were reared in floor pens with a 2‐in layer of clean, dry litter material, and each replicate was housed separately to avoid cross‐contamination. During the first week of brooding, an initial temperature of 32–34°C was maintained using infrared heat lamps. The temperature was gradually reduced by 2–3°C per week to reach approximately 24°C by the end of the third week, in accordance with standard brooding protocols. Continuous lighting (24 h) was provided during the first 3 days to help chicks adjust to the environment, followed by 23 h of light and 1 h of darkness throughout the remainder of the trial. Adequate ventilation and controlled environmental conditions were ensured in an open‐sided poultry house to maintain bird comfort. All birds were fed diets formulated according to NRC (1994) recommendations, with a starter diet provided during the starter phase (1–10 days), a grower diet during the grower phase (11–28 days) and a finisher diet during the finisher phase (29–42 days) as shown in Table 1. All birds were vaccinated according to standard protocols: Newcastle disease (ND) vaccine was administered via eye drop at 5 and 21 days of age, and infectious bursal disease (IBD) vaccine was given at 12 and 24 days of age via drinking water. The experimental trial lasted for a total of 6 weeks. The control group received a basal diet without any additives. The remaining four groups were supplemented as follows: the prebiotic group received 200 mg/kg of a prebiotic (MOS); the garlic extract group received 60 mg/kg of garlic extract; the Combo‐I group received a combination of 100 mg/kg prebiotic and 30 mg/kg garlic extract (i.e., 50% of the individual doses); and the Combo‐II group received the full doses of both supplements—200 mg/kg prebiotic and 60 mg/kg garlic extract for a period of 42 days including 1 week of adaptation. Feed and fresh drinking water were provided ad libitum, and separate feeders and drinkers were assigned to each replicate. All birds were managed under standard commercial conditions with adherence to routine biosecurity, hygiene and vaccination (all major infectious diseases including ND, IBD, infectious bronchitis [IB] and avian influenza), protocols to ensure flock health and consistency across treatments.

**TABLE 1:** Broiler diet formulations and nutrient composition (NRC 1994).

Ingredients (%)	Starter (1–10 days)	Grower (11–28 days)	Finisher (29–42 days)
Maize	52.00	55.00	60.00
Soybean meal (44%)	38.00	35.00	30.00
Vegetable oil	2.50	3.00	3.50
Dicalcium phosphate	1.80	1.60	1.40
Limestone	1.00	1.00	1.00
dl‐methionine	0.25	0.20	0.15
l‐lysine HCl	0.20	0.15	0.10
Salt	0.30	0.30	0.30
Vitamin‐mineral premix^a^	0.50	0.50	0.50
Total	100.00	100.00	100.00

Calculated chemical composition			
Nutrient	Starter (1–10 days)	Grower (11–28 days)	Finisher (29–42 days)
Metabolizable energy (kcal/kg)	3000.00	3100.00	3200.00
Crude protein (%)	22.00	20.00	18.00
Calcium (%)	1.00	0.90	0.85
Available phosphorus (%)	0.45	0.40	0.35
Lysine (%)	1.20	1.05	0.90
Methionine + cystine (%)	0.90	0.80	0.70

^a^Vitamin‐mineral premix provides per kg of diet: vitamin A (12,000 IU), D3 (2000 IU), E (10 mg), K (3 mg), B1 (2 mg), B2 (6 mg), B6 (3 mg), B12 (0.02 mg), niacin (40 mg), folic acid (1 mg), pantothenic acid (10 mg), biotin (0.1 mg), choline chloride (500 mg), iron (60 mg), zinc (60 mg), manganese (60 mg), copper (5 mg), iodine (1 mg) and selenium (0.2 mg).

### 2.2 Preparation of Prebiotic and Garlic Extract

Garlic extract was prepared from dried cloves (Al‐Kabeer Pharmaceuticals, Pakistan) through ethanol extraction, filtration and evaporation. The extract contained allicin (∼1.5 mg/g), diallyl disulphide (∼0.8 mg/g), S‐allyl cysteine (∼0.5 mg/g) and ajoene (∼0.3 mg/g), as identified by chromatographic analysis. The prebiotic used was MOS (Bio‐MOS, Alltech, USA), derived from *Saccharomyces cerevisiae* cell walls and known for enhancing gut health. Both supplements were added to the basal diet at the designated concentrations for each treatment.

### 2.3 Zootechnical Parameters

All performance indicators, including feed intake, BWG and feed conversion ratio (FCR), were systematically recorded. At the end of the 42‐day trial, two birds per replicate were randomly selected and slaughtered to assess carcass traits. Birds were weighed, defeathered and eviscerated, and carcass weight was recorded. Dressing percentage was calculated as the ratio of carcass weight to live body weight multiplied by 100. Organ weights, including liver, heart and gizzard, were expressed as a percentage of live body weight. Abdominal fat was removed and weighed, also expressed as a percentage of live weight. Data collected included dressing percentage, carcass weight (g), liver %, heart %, gizzard % and abdominal fat %.

### 2.4 Antibody Titres

On Day 1, prior to administration of any dietary treatments or vaccinations, baseline blood samples (1 mL) were collected (Almahallawi et al. 2024) from five randomly selected chicks per treatment group to assess maternal antibody levels. Serum was separated and analysed for ND and IBD antibody titres to establish initial immunity status. Additionally, on Day 30, 1 mL of blood was collected from five birds per replicate for post‐vaccination antibody titre assessment. ND antibody titres were determined using the haemagglutination inhibition (HI) test. HI titres were recorded as the reciprocal of the highest serum dilution that inhibited haemagglutination, and results were expressed as log_2_X (Alqahtani et al. 2024).

IBD antibody titres were measured using a commercial indirect ELISA kit (IDEXX, USA), following the manufacturer's instructions. Briefly, serum samples were diluted and added to ELISA plates pre‐coated with IBDV antigen. Plates were incubated at 37°C for 30–60 min, washed to remove unbound antibodies and then incubated with enzyme‐conjugated secondary antibodies. After a final wash, a chromogenic substrate was added, and colour development was measured using an ELISA reader at 450 nm. Antibody titres were calculated using a standard curve or software provided with the kit.

### 2.5 Immunoglobulin Estimation

Fifteen millilitre blood from sheep was collected in EDTA tubes, washed with PBS, and SRBCs were prepared at 0.25% in PBS. At 21 days, birds were injected with 0.1 mL SRBCs. After 14 days, blood was collected, serum isolated and antibody titres measured. A direct haemagglutination assay was used to measure total antibodies (IgM, IgA and IgG). Serum was heat‐inactivated (56°C, 30 min), serially diluted in PBS–BSA and mixed with 1% SRBCs. Plates were incubated (37°C, 24 h), and titres read at 50% agglutination. For IgM/IgG differentiation, serum was treated with 0.2 M 2‐mercaptoethanol (2‐ME, 37°C, 30 min). Haemagglutination post‐treatment indicated IgG; the difference from total titre represented IgM (Hina et al. 2024).

### 2.6 Gut Morphology and Histological Procedure

At the end of the experimental period, feed was withdrawn overnight from selected birds. The birds were weighed and humanely sacrificed. A 2‐cm segment from the midpoint of the duodenum was excised, rinsed with physiological saline and preserved in 10% neutral buffered formalin. Tissue samples were processed and stained following the standard protocol. Fixed tissues were dehydrated, embedded in paraffin, sectioned at 5 µm thickness using a microtome, mounted on glass slides and stained with hematoxylin and eosin (H&E). Villus height (VH) and CD were measured using a light microscope under calibrated magnifications (Purwanti et al. 2024). A minimum of 10 well‐oriented villi and crypts per sample were evaluated to obtain mean values.

### 2.7 Gut Microbiota Evaluation

On Day 42, two birds per replicate were randomly selected and humanely euthanized by cervical dislocation in accordance with institutional ethical guidelines. Ileal contents of approximately 1 g were aseptically collected and homogenized in 9 mL of sterile 1% peptone solution (1:10 dilution). The homogenates were serially diluted, and appropriate dilutions were plated on selective agar media specific for each bacterial species. *Lactobacillus* spp. were cultured on de Man, Rogosa and Sharpe (MRS) agar and incubated at 37°C with 5% CO_2_ for 24 h, whereas *Escherichia coli* and *Salmonella* were plated on MacConkey agar and incubated at 37°C for 24 h. After incubation, bacterial colonies were counted using a colony counter, and microbial populations were expressed as log_10_ colony‐forming units (CFU/g) of ileal digesta. All samples and dilutions were plated in triplicate to ensure accuracy and reproducibility (Al‐Khalaifah et al. 2025).

### 2.8 Statistical Analysis

All data were analysed using one‐way analysis of variance (ANOVA) under a completely randomized design (CRD). Treatment means were compared using Tukey's Honest Significant Difference (HSD) test at a significance level of *p* < 0.05. Data were expressed as means ± standard error of the mean (SEM). Statistical analyses were conducted using SPSS software (version 21).

## 3 Results

The supplementation of garlic extract, MOS and their combinations significantly influenced broiler performance across all growth phases (Table 2). During the starter phase (1–10 days), birds in the Combo‐I group exhibited significantly higher BWG (*p* = 0.0356) and improved FCR (*p* = 0.0318) compared to the control and other treatments, whereas feed intake differences were not statistically significant (*p* = 0.0845). In the grower phase (11–28 days), Combo‐I again led to the greatest weight gain (*p* = 0.0425) and most efficient FCR (*p* = 0.0383), with feed intake also differing significantly among groups (*p* = 0.0452). Similar trends continued in the finisher period (29–42 days), where Combo‐I showed superior performance with the highest weight gain and lowest FCR (*p* < 0.05), although feed intake varied significantly as well (*p* = 0.0458). Overall, Combo‐I (a 50:50 blend of garlic extract and MOS) consistently improved growth metrics and feed efficiency throughout the trial, indicating a synergistic benefit of the combined supplementation strategy.

**TABLE 2:** Effect of prebiotic, garlic and their combination on production performance in broiler chicks.

Period	Treatments	Feed intake (g/bird)	Body weight gain (g/bird)	Feed conversion ratio (FCR)
Starter period (g/week) 1–10 days	Control (basal diet)	240.68^b^	162.58^d^	1.48^a^
	Prebiotic	230.85^c^	181.54^bc^	1.27^bc^
	Garlic extract	245.48^b^	183.85^bc^	1.33^b^
	Combo‐I (50:50)	242.84^b^	201.71^a^	1.20^c^
	Combo‐II (100:100)	260.45^a^	173.54^b^	1.50^a^
	*p* value	0.0845	0.0356	0.0318
	SEM	3.142	4.441	0.0213
Grower period (g/week) 11–28 days	Control (basal diet)	1265.47^ab^	975.72^c^	1.29^a^
	Prebiotic	1275.48^a^	991.60^b^	1.28^a^
	Garlic extract	1260.47^b^	997.68^b^	1.26^ab^
	Combo‐I (50:50)	1240.58^c^	1020.54^a^	1.21^b^
	Combo‐II (100:100)	1280.54^a^	995.52^b^	1.28^a^
	*p* value	0.0452	0.0425	0.0383
	SEM	5.442	10.52	0.251
Finisher period (g/week) 29–42 days	Control (basal diet)	2430.51^c^	1032.47^c^	2.35^a^
	Prebiotic	2450.22^b^	1058.54^b^	2.31^b^
	Garlic extract	2442.84^c^	1052.41^b^	2.32^b^
	Combo‐I (50:50)	2410.51^d^	1120.34^a^	2.15^c^
	Combo‐II (100:100)	2464.62^a^	1059.25^b^	2.32^b^
	*p* value	0.0458	0.0469	0.0343
	SEM	13.25	17.54	0.307

*Note*: The means within the same column with at least one common letter do not have significant difference (*p* > 0.05). Control (basal diet): broilers fed a standard diet without additives. Prebiotic: broilers fed basal diet supplemented with 200 mg/kg mannan oligosaccharide (MOS). Garlic extract: broilers fed basal diet supplemented with 60 mg/kg garlic extract. Combo‐I (50:50): broilers fed basal diet with 50% of both prebiotic (100 mg/kg) and garlic extract (30 mg/kg). Combo‐II (100:100): broilers fed basal diet with full doses of both prebiotic (200 mg/kg) and garlic extract (60 mg/kg).

Abbreviation: SEM, standard error of the means.

The effects of dietary supplementation of prebiotic, garlic extract and their combinations on carcass characteristics are presented in Table 3. The treatments had a significant effect on carcass weight (*p* = 0.03), with Combo‐II showing the highest weight. Dressing percentage, liver %, heart %, gizzard % and abdominal fat % were not significantly affected (*p* > 0.05), indicating that the additives did not negatively influence overall carcass composition.

**TABLE 3:** Carcass characteristics of broiler fed prebiotic and garlic.

	Dressing (%)	Carcass weight (g)	Liver (%)	Heart (%)	Gizzard (%)	Abdominal fat (%)
Control (basal diet)	72	1748	2.0	0.5	2.5	1.8
Prebiotic	72	1789	2.1	0.5	2.6	1.7
Garlic extract	73	1783	2.0	0.5	2.5	1.6
Combo‐I (50:50)	74	1785	2.1	0.5	2.4	1.5
Combo‐II (100:100)	73	1798	2.1	0.5	2.5	1.6
*p* value	0.55	0.03	0.09	0.17	0.43	0.76
SEM	0.1	50.4	0.01	0.01	0.01	0.2

*Note*: The means within the same column with at least one common letter do not have significant difference (*p* > 0.05). Control (basal diet): broilers fed a standard diet without additives. Prebiotic: broilers fed basal diet supplemented with 200 mg/kg mannan oligosaccharide (MOS). Garlic extract: broilers fed basal diet supplemented with 60 mg/kg garlic extract. Combo‐I (50:50): broilers fed basal diet with 50% of both prebiotic (100 mg/kg) and garlic extract (30 mg/kg). Combo‐II (100:100): broilers fed basal diet with full doses of both prebiotic (200 mg/kg) and garlic extract (60 mg/kg).

Abbreviation: SEM, standard error of the means.

Dietary treatments significantly influenced antibody titres against both ND and IBD (*p* = 0.0345 and *p* = 0.0341, respectively), as shown in Table 4. Broilers receiving the Combo‐I diet exhibited the highest ND and IBD antibody responses, followed by the Combo‐II group, indicating a synergistic immunostimulatory effect of garlic extract and MOS. The garlic extract and prebiotic groups also showed elevated antibody levels compared to the control, but these were significantly lower than the combination treatments. Notably, no mortality was recorded in any treatment group throughout the study period (*p* = 0.000), indicating that all dietary regimens were safe and well‐tolerated.

**TABLE 4:** Effect of prebiotic, probiotic and their combination on antibody titre and mortality per cent in broiler chicks.

Treatments	ND	IBD	Mortality per cent
Control (basal diet)	4.5^c^	2196.15^c^	0.00
Prebiotic (200 mg/kg 0.03 mL/kg)	4.1^bc^	2689.61^b^	0.00
Garlic extract (60 mg/kg basal diet)	5.4^b^	2568.38^b^	0.00
Combo‐I (50:50)	6.8^a^	3574.36^a^	0.00
Combo‐II (100:100)	5.8^ab^	3357.82^ab^	0.00
*p* value	0.0345	0.0341	0.000
SEM	1.131	4.953	0.000

*Note*: The means within the same column with at least one common letter do not have significant difference (*p* > 0.05). Control (basal diet): broilers fed a standard diet without additives. Prebiotic: broilers fed basal diet supplemented with 200 mg/kg mannan oligosaccharide (MOS). Garlic extract: broilers fed basal diet supplemented with 60 mg/kg garlic extract. Combo‐I (50:50): broilers fed basal diet with 50% of both prebiotic (100 mg/kg) and garlic extract (30 mg/kg). Combo‐II (100:100): broilers fed basal diet with full doses of both prebiotic (200 mg/kg) and garlic extract (60 mg/kg).

Abbreviations: IBD, infectious bursal disease; ND, Newcastle disease; SEM, standard error of the means.

As shown in Table 5, supplementation with prebiotic, garlic extract and their combinations significantly influenced the serum immunoglobulin levels (*p* < 0.05 for IgM and IgA; *p* = 0.0501 for IgG). Birds receiving the Combo‐I and Combo‐II diets exhibited notably higher titres of IgM, IgA and IgG compared to the control group. Among all groups, Combo‐I resulted in the highest levels of all three immunoglobulins, suggesting enhanced humoral immunity due to the synergistic action of both supplements. Although garlic extract and prebiotic groups also increased antibody levels compared to control, the effect was less pronounced than in the combination treatments.

**TABLE 5:** Effect of prebiotic, probiotic and their combination on immunoglobulin titre against the sheep RBCs in broiler chicks.

Treatments	IgM	IgA	IgG
Control (basal diet)	1.36^c^	0.02^c^	0.03^b^
Prebiotic (200 mg/kg 0.03 mL/kg)	1.63^bc^	0.08^b^	0.04^b^
Garlic extract (60 mg/kg basal diet)	1.69^b^	0.09^b^	0.04^b^
Combo‐I (50:50)	2.13^a^	0.18^a^	0.09^a^
Combo‐II (100:100)	2.11^a^	0.14^a^	0.08^a^
*p* value	0.0213	0.0332	0.0501
SEM	0.068	0.075	0.021

*Note*: The means within the same column with at least one common letter do not have significant difference (*p* > 0.05). Control (basal diet): broilers fed a standard diet without additives. Prebiotic: broilers fed basal diet supplemented with 200 mg/kg mannan oligosaccharide (MOS). Garlic extract: broilers fed basal diet supplemented with 60 mg/kg garlic extract. Combo‐I (50:50): broilers fed basal diet with 50% of both prebiotic (100 mg/kg) and garlic extract (30 mg/kg). Combo‐II (100:100): broilers fed basal diet with full doses of both prebiotic (200 mg/kg) and garlic extract (60 mg/kg).

Abbreviation: SEM, standard error of the means.

**FIGURE 1:**
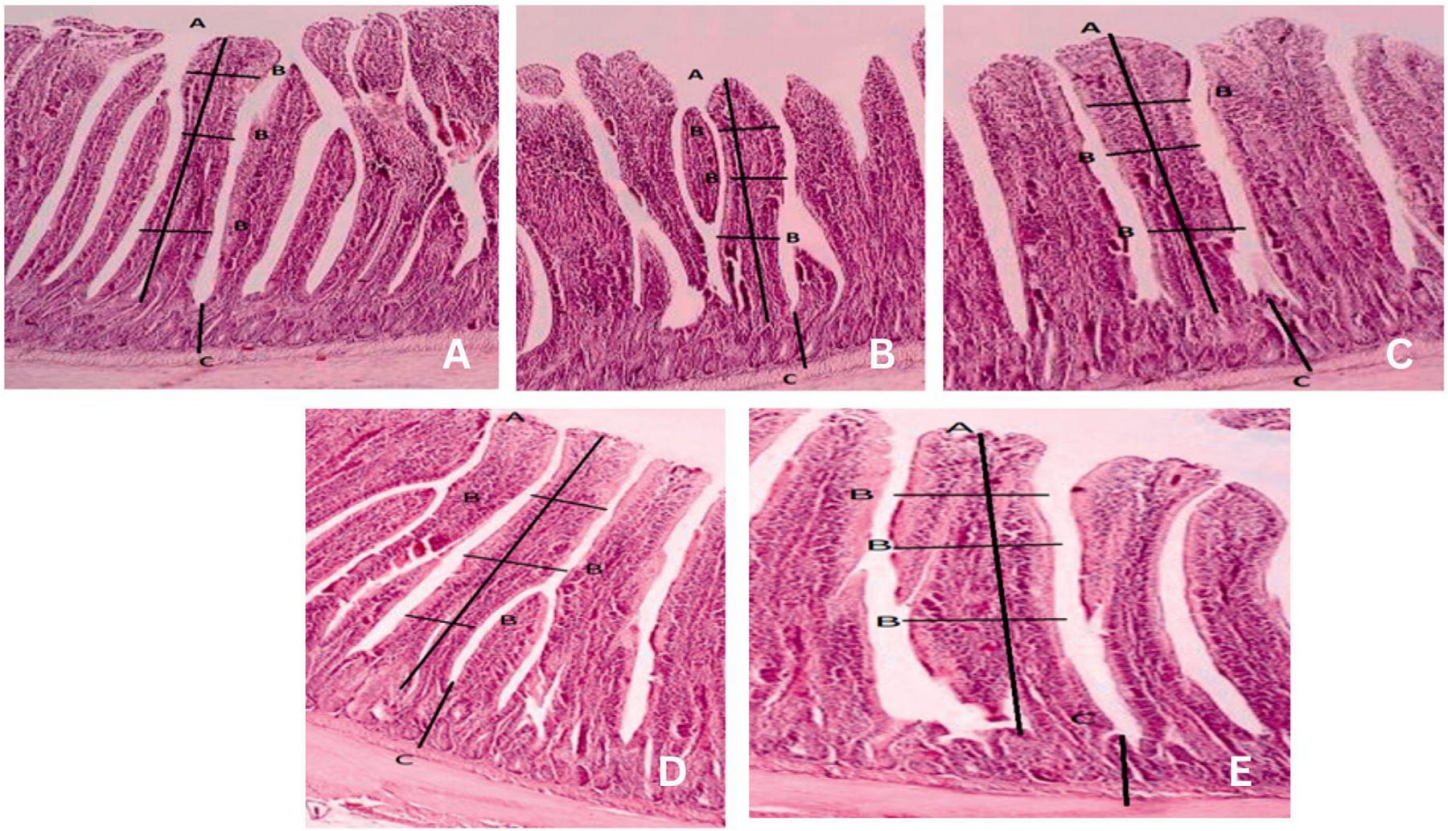
Duodenum villus morphology in control group (A), at 40×, garlic extract‐fed group (B), prebiotic‐fed group (C), Combo‐I group (D), and Combo‐II group (E).

Duodenal histomorphological parameters were significantly influenced by dietary treatments (*p* < 0.05; Table 6 and Figure 1). Birds in the Combo‐I and Combo‐II groups exhibited the most prominent improvements, characterized by greater villus height, wider villi and shallower CD, resulting in a significantly higher VH:CD ratio compared to other groups. In contrast, the control group had the shortest villi and deepest crypts, resulting in the lowest VH:CD ratio. Prebiotic and garlic extract groups showed intermediate effects, with moderate enhancements in villus structure relative to the control. These morphological changes suggest improved absorptive capacity and gut health in birds receiving combined supplementation.

**TABLE 6:** Effect of prebiotic, probiotic and their combination on duodenum in broiler chicks.

Treatments	Villus height (µm)	Villus width (µm)	Crypt depth (µm)	VH:CD
Control (basal diet)	570.34^d^	45.34^c^	110.47^a^	5.16^c^
Prebiotic (200 mg/kg 0.03 mL/kg)	640.41^c^	67.31^b^	81.54^c^	7.85^b^
Garlic extract (60 mg/kg basal diet)	689.35^b^	66.67^b^	92.34^b^	7.46^b^
Combo‐I (50:50)	763.62^a^	105.21^a^	73.56^d^	10.38^a^
Combo‐II (100:100)	751.42^a^	99.56^a^	70.34^d^	10.68^a^
*p* value	0.0223	0.0237	0.0352	0.0325
SEM	27.18	5.52	6.58	0.646

*Note*: Control (basal diet): broilers fed a standard diet without additives. Prebiotic: broilers fed basal diet supplemented with 200 mg/kg mannan oligosaccharide (MOS). Garlic extract: broilers fed basal diet supplemented with 60 mg/kg garlic extract. Combo‐I (50:50): broilers fed basal diet with 50% of both prebiotic (100 mg/kg) and garlic extract (30 mg/kg). Combo‐II (100:100): broilers fed basal diet with full doses of both prebiotic (200 mg/kg) and garlic extract (60 mg/kg).

Abbreviation: VH, villus height.

As shown in Table 7, supplementation of broiler diets with prebiotic, garlic extract and their combinations influenced ileal bacterial counts. Both single and combined treatments non‐significantly (*p* > 0.05) reduced pathogenic bacteria, *E. coli* and *Salmonella*, while enhancing (*p* > 0.05) the population of beneficial *Lactobacillus* spp. The combination treatments were more effective than individual supplements in modulating the gut microflora.

**TABLE 7:** Effect of prebiotic, garlic extract and their combination on ileal bacterial counts (log10 CFU/g) in broiler chickens (log10 CFU/g).

Treatments	*Escherichia coli*	*Salmonella*	*Lactobacillus* spp.
Control (basal diet)	5.81	4.62	7.32
Prebiotic (200 mg/kg 0.03 mL/kg)	5.68	4.45	7.45
Garlic extract (60 mg/kg basal diet)	5.65	4.40	7.43
Combo‐I (50:50)	5.51	4.26	7.54
Combo‐II (100:100)	5.48	4.18	7.52
*p* value	0.003	0.002	0.0004
SEM	0.15	0.44	0.06

*Note*: Control (basal diet): broilers fed a standard diet without additives. Prebiotic: broilers fed basal diet supplemented with 200 mg/kg mannan oligosaccharide (MOS). Garlic extract: broilers fed basal diet supplemented with 60 mg/kg garlic extract. Combo‐I (50:50): broilers fed basal diet with 50% of both prebiotic (100 mg/kg) and garlic extract (30 mg/kg). Combo‐II (100:100): broilers fed basal diet with full doses of both prebiotic (200 mg/kg) and garlic extract (60 mg/kg).

Abbreviation: CFU, colony‐forming units.

## 4 Discussion

The present study demonstrated that a 50:50 combination of garlic extracts and prebiotics (Combo‐I) significantly improved broiler performance across all growth phases, outperforming individual treatments and other combinations in terms of BWG and FCR. These findings align with recent research highlighting the synergistic effects of combining functional feed additives to enhance nutrient utilization, gut health and overall growth performance (Ferdous et al. 2019). During the starter phase (1–10 days), Combo‐I‐fed birds exhibited the highest BWG and best FCR despite moderate feed intake. This suggests early stimulation of gut development and improved nutrient digestibility, likely due to enhanced enzyme secretion and favourable microbial colonization (Rubio 2019). Prebiotics promote beneficial bacterial populations such as *Lactobacillus* and *Bifidobacteria*, which in turn enhance intestinal barrier integrity and metabolite production (short‐chain fatty acids [SCFAs]), contributing to improved feed efficiency (Shehata et al. 2022). Garlic extracts exert antimicrobial and anti‐inflammatory effects, which play a key role in supporting early immune development and gut functionality. The antimicrobial compounds, such as allicin and diallyl disulphide, help reduce pathogenic bacterial populations in the gut, thereby lowering the risk of infections and maintaining a balanced microbiota. This reduction in pathogenic load allows the immune system to develop more effectively, enhancing both innate and adaptive immune responses. The anti‐inflammatory compounds in garlic, including S‐allyl cysteine and ajoene, help modulate excessive inflammatory reactions in the intestinal mucosa, preserving tissue integrity and promoting nutrient absorption. Together, these effects improve gut barrier function, enhance digestion and nutrient utilization and support overall growth and health in poultry (Ullah et al. 2022; Imtiaz et al. 2023). In the grower phase (11–28 days), Combo‐I again led to superior BWG and FCR, with relatively lower feed intake, indicating improved feed efficiency. This is consistent with findings by Ferdous et al. (2019), who reported that combinations of garlic extracts and prebiotics improved gut morphology (e.g., VH and CD) and nutrient transporter expression, leading to better growth performance. The garlic extract group alone also showed enhanced feed intake and weight gain, likely due to bioactive compounds (e.g., flavonoids and essential oils) stimulating appetite and digestive enzyme secretion (Hafeez et al. 2021). In the finisher phase (29–42 days), Combo‐I birds maintained their growth advantage with the highest BWG and lowest FCR, even with the lowest feed intake, reinforcing the additive's efficiency. Conversely, the Combo‐II (100:100) group had the highest feed intake but did not achieve proportionate weight gain, suggesting that excessive dosing may reduce synergistic benefits or lead to metabolic imbalance.

In the current study, while dressing percentage, liver %, heart %, gizzard % and abdominal fat % were not significantly altered (*p* > 0.05), carcass weight was significantly influenced by the treatments (*p* = 0.03), with Combo‐II showing the highest carcass weight. This indicates that the improved growth performance of birds, particularly in the Combo‐I and Combo‐II groups, translated into heavier carcasses without negatively affecting organ proportions or fat deposition. The alignment of superior BWG and FCR with increased carcass weight underscores the effectiveness of these additive combinations in promoting both growth efficiency and desirable meat yield.

The highest ND and IBD antibody titres were observed in birds fed the Combo‐I (50:50) blend, indicating a synergistic interaction between the two additive types. The improved ND and IBD titres in Combo‐I and Combo‐II groups suggest an enhanced adaptive immune response, likely mediated through gut–immune axis modulation. Prebiotics stimulate the proliferation of beneficial gut microbiota (*Lactobacillus* and *Bifidobacteria*), which, in turn, produce SCFAs—key modulators of immune cell differentiation and function (Liu et al. 2023). These SCFAs support the maturation of GALT and enhance antigen‐presenting cell activity, leading to stronger vaccine responsiveness (Kogut 2022). Garlic extracts contribute through bioactive compounds such as polyphenols, flavonoids and essential oils, which exhibit antioxidant, anti‐inflammatory and antimicrobial activities. These compounds support lymphocyte proliferation, cytokine production and antibody synthesis (Khan et al. 2012; Dabool et al. 2024). Mechanistically, the combined action of MOS and garlic bioactives may enhance immune function by reinforcing mucosal barrier integrity, reducing oxidative stress and modulating toll‐like receptor (TLR) signalling. MOS promotes the growth of beneficial gut microbes (*Lactobacillus* and *Bifidobacteria*), which produce short‐chain fatty acids that regulate immune cell differentiation and mucosal defence (Liu et al. 2023). It also upregulates TLR2 and TLR4 expression, improving antigen presentation and epithelial barrier function (Cario et al. 2004). Garlic bioactives, such as allicin, polyphenols and flavonoids, exhibit antioxidant and immunostimulatory effects, enhancing lymphocyte proliferation, cytokine secretion and antibody synthesis (Khan et al. 2012). Together, microbiota‐mediated modulation from MOS and direct immunostimulatory effects of garlic phytochemicals synergistically enhanced ND and IBD antibody titres in the Combo‐I group.

The highest concentrations of IgM and IgG in the Combo‐I group suggest improved systemic immune responsiveness, whereas elevated IgA levels reflect enhanced mucosal immunity, particularly within the gut—the first line of defence against enteric pathogens. Prebiotics enhance IgA production by supporting plasma cell differentiation in Peyer's patches and stimulating cytokines such as interleukin‐6 and transforming growth factor‐β, which drive class switching to IgA (Rousseaux et al. 2023). Garlic extracts, especially those rich in terpenoids and phenolic acids, have been reported to stimulate macrophage and dendritic cell activity, leading to increased B‐cell activation and subsequent immunoglobulin synthesis (Khan et al. 2012). The synergism observed in Combo‐I and Combo‐II could be attributed to complementary mechanisms, wherein prebiotics improve gut microbial balance and immune priming, whereas garlic extracts directly stimulate immune signalling pathways. Similar improvements in antibody titres and immunoglobulin levels have been reported with phytogenic and prebiotic supplementation. For example, Elbaz et al. (2021) observed higher immune response in broilers fed a synbiotic blend. Likewise, Gadde et al. (2017) showed that phytogenic compounds improved immune organ development and vaccine response. However, the current findings emphasize that the enhanced immune parameters in the Combo‐I group likely result from an optimal balance of prebiotic and phytogenic components rather than higher inclusion rates alone. Future studies should focus on titration trials to identify the most effective combination ratio and dosage range that sustain immune stimulation without inducing metabolic stress or nutrient imbalance.

The greatest villus height and width in Combo‐I and Combo‐II groups reflect a larger absorptive surface area, which facilitates more efficient nutrient uptake. VH is positively correlated with the maturity and functional capacity of enterocytes, while increased width reflects greater epithelial surface density (Akram et al. 2025). The synergistic effects observed with the combination groups may be due to modulation of the gut microbiota and reduction of intestinal inflammation, both of which contribute to optimal epithelial development. Prebiotics, particularly oligosaccharides, selectively stimulate the growth of beneficial microbiota, which in turn produce SCFAs like butyrate. Butyrate promotes enterocyte proliferation and differentiation, thereby increasing villus height and nutrient transport capacity (Kogut 2022). Garlic extracts contain bioactive compounds such as flavonoids and essential oils, which possess anti‐inflammatory and antimicrobial properties. These reduce pathogen‐induced damage and oxidative stress in the gut epithelium, leading to healthier, more developed villi (Khan et al. 2012). The significantly lower CD observed in Combo‐I and Combo‐II groups indicates a reduction in intestinal epithelial turnover, suggesting lower immune stress and reduced pathogen load (Gadde et al. 2017). A shallow CD, combined with taller villi, results in a higher VH:CD ratio, which is widely regarded as a marker of improved intestinal integrity and absorptive efficiency (Islam et al. 2024). This effect is likely due to the combined action of prebiotics improving microbial balance and garlic extracts enhancing barrier function by upregulating tight junction proteins and reducing proinflammatory cytokines. Together, they create a more stable and functional intestinal environment.

Previous studies support these observations. For instance, Elbaz et al. (2021) found that the use of phytogenic compounds or prebiotics alone improved villus architecture and intestinal health, but combined supplementation yielded greater effects. Combo‐I (50:50) was especially effective in the current study, suggesting an optimal dosage balance that maximizes the additive benefits without overloading the gut with bioactives or fermentable substrates. Additionally, Ferdous et al. (2019) and Shehata et al. (2022) observed similar synergistic effects on duodenal morphology with synbiotic inclusion, linking these changes to enhanced growth performance and feed efficiency—as was observed concurrently in this study. Beyond productivity gains, the replacement of antibiotics with natural supplements like MOS and garlic extract can reduce the risk of antimicrobial resistance, lower environmental antibiotic residues and minimize ecological disruption in soil and water systems. Economically, the use of natural additives sourced from renewable materials may offer long‐term cost benefits by improving feed efficiency, reducing disease incidence and aligning with consumer demand for antibiotic‐free poultry products.

## 5 Conclusion

In conclusion, the combination of 100 mg/kg prebiotic (MOS) and 30 mg/kg garlic extract (Combo‐I) significantly improved broiler growth performance, immune response and intestinal morphology compared with individual supplementation. This indicates a synergistic interaction between prebiotic and garlic extract, where even at half doses their combined effect surpassed the full individual treatments. Such findings highlight the potential of integrating natural feed additives in poultry diets as sustainable and effective alternatives to AGPs, contributing to enhanced productivity, improved gut health and safer poultry production systems.

## Author Contributions

**Azizullah Khan**: investigation, methodology. **Muhammad Mushtaq**: conceptualization, investigation, data curation. **Muqadar Shah**: project administration, supervision. **Rifat Ullah Khan**: methodology, formal analysis. **Shabana Naz**: writing – original draft, writing – review and editing. **Ala Abudabos**: writing – original draft, writing – review and editing. **Muhammad Israr**: software, statistical analysis. **Rasha Alonaizan**: data curation, validation.

## Funding

The authors have nothing to report.

## Ethics Statement

This study was approved by the ethical committee of the Department of Poultry Science, Faculty of Animal Husbandry and Veterinary Sciences, The University of Agriculture Peshawar, Pakistan under notification No. L‐ 452/AH/UAP dated 16/11/2022.

## Conflicts of Interest

The authors declare no conflicts of interest.

## Data Availability

Data is available from the corresponding author upon reasonable request.
